# Compassionate care of nurses for the elderly admitted to the COVID-19 wards in teaching hospitals of southern Iran

**DOI:** 10.1186/s12912-023-01670-6

**Published:** 2024-01-02

**Authors:** Fereshte Faghihi, Ladan Zarshenas, Banafsheh Tehranineshat

**Affiliations:** 1 Department of Nursing, School of Nursing and Midwifery, Student Research Committee, Shiraz University of Medical Sciences, Shiraz, Iran; 2 Department of Nursing, School of Nursing and Midwifery, Shiraz University of Medical Sciences, Shiraz, Iran; 3 Department of Nursing, Faculty of Nursing and Midwifery, Hormozgan University of Medical Sciences, Bandar Abbas, Iran

**Keywords:** Compassion, Nursing care, Patients, Elderly, COVID-19

## Abstract

**Background:**

Compassionate care is the main indicator of the quality regarding nursing care. The importance of this care in the recovery process for the elderly hospitalized for COVID-19 has been under-researched in studies. Therefore, this study aimed to determine the compassionate care level of nurses to the elderly hospitalized in the COVID-19 wards of teaching hospitals in the south of Iran.

**Methods:**

This descriptive-analytical study was conducted on 212 nurses working in the COVID-19 wards of teaching hospitals in the south of Iran, who were selected through census in a cross-sectional study. The data were collected using the Tehranineshat et al. nurses’ compassionate care questionnaire and then the data were analyzed using descriptive and analytical statistics along with SPSS software version 22.

**Results:**

The mean score of nurses’ compassionate care was 130.18 ± 9.42, which was at a high level. The highest and lowest scores were related to professional performance (43.17 ± 2.799) and empathic communication dimension (27.76 ± 2.970). No significant relationship was found between variables such as gender, marital status, education, work experience, and job position with the compassionate care score (P > 0.05).

**Conclusion:**

Nurses providing care for hospitalized elderly are recommended to consider all aspects of compassionate care, especially empathic communication, in their educational planning.

## Background

Ageing populations are increasing in all countries, including Iran, which is transforming the world’s population growth and health charts [1]. According to predictions, the elderly population will increase from 800 million people in the world to two billion people by 2050, which includes both developed and developing countries [2]. Although chronic diseases are common in the elderly, COVID-19 as an acute disease on the health and economic situation in the world has caused many victims, especially among the elderly. Statistics show that most of the deaths caused by Covid-19 are in countries such as the United States, Italy, Spain, France, and the United Kingdom, which have a high elderly population and often suffer from life-threatening infections and require long-term care [3].

The elderly are more prone to contracting Covid-19 due to various reasons, including a low immune system, chronic diseases that can mask the symptoms of infection, and the use of several types of medication. Further, factors such as lack of attention or lack of ability to observe the principles of personal hygiene and self-care lead fully and correctly to the exacerbation of the risk of the elderly suffering from this disease. On the other hand, loneliness and lack of sufficient support from other family members, simplistic treatment of diseases and relating all symptoms to old age, lack of visiting or visiting late, unwillingness to take medicine, improper nutrition, and forgetfulness make these people among the most vulnerable sections of society in the face of the COVID-19 [4].

This vulnerable group needs to be attended to physically, psychologically, and in terms of their care needs. The health care provided to these people should not only improve the chances of their contracting COVID-19, but also enhance the quality of care provided at the time of their infection and hospitalization. Compassionate care is one of the main approaches to providing supportive care for this group during hospitalization [5]. Compassion and compassionate care are considered as the main elements in nursing [6]. According to studied, compassion has been defined as “awareness of others’ pain and suffering and action to for its prevention or elimination” [7–9]. Nurses play an important role in meeting the complex needs of the elderly in the crisis and it is essential to use specialist nurses for addressing the physical and psychological needs of this high-risk population [10]. Speaking kindly, actively listening to the patient and his family, and building a relationship based on trust are other features of compassionate care that can alleviate the suffering of a patient with COVID-19. Compassion can be done with small actions such as smiling at the patient, effective support, and providing necessary information [11]. Leamy et al. stated that small acts of kindness can make unbearable situations and problems bearable. Compassion can lead to a better regulation of the body’s physiological system, which directly related to the reduction of the pituitary-hypothalamus-adrenal axis reaction, cardiovascular reaction, and cortisol reaction, the increase of the parasympathetic nervous system reaction and immune system, which reduce stress in the patients [12]. Based on the results of a study from the perspective of hospitalized elderly, compassion and empathy were essential components of nursing care. Hospitalization during the period of COVID-19 caused the elderly to lose their sense of independence and control, and receiving care with compassion was a positive and pleasant experience during hospitalization and suffering from COVID-19 [13]. From the point of view of hospitalized elderly in another study, being empathetic, open minded, helpful, having a friendly attitude of a nurse was one of the examples of compassionate care during the COVID-19 pandemic. It was important for nurses to look at hospitalized elderly patients as human beings with feelings and emotions [14].

Abozied et al. (2020) studied perception of elderly patients about compassionate care by nurses. They concluded that the elderly patients on which they studied had a high perception and positive view about compassionate care; their responses were influenced by education and their duration of hospital stay. That study recommended that the opinions of elderly patients about compassion-based nursing care be investigated regularly [15].

In another study to explore the “effectiveness of compassion-based therapy on sense of loneliness and cognitive resilience of elderly men” on 30 elderly men aged 60–70 years old, it was found that there was a significant difference between the intervention and control groups regarding sense of loneliness and cognitive resilience. In other words, compassionate treatment led to diminished sense of loneliness and enhanced resilience of the elderly [16].

Compassionate care involves actively listening to a patient’s story and empathizing with their perspective, particularly when dealing with elderly patients. This approach leads to a better understanding of the patient’s specific needs and increases the level of compassionate care provided. As a result, there is a growing need to prioritize the development of nurses’ competencies in compassionate care through their pre- and postgraduate training [14].

Compassionate care is important in improving the quality of care for elderly patients and high-quality nursing care when these people are infected and admitted to the hospital, especially during the COVID-19 era. This study aimed to evaluate the compassionate care of nurses in caring for the elderly in the COVID-19 wards in teaching hospitals located in the south of Iran in 2021–2022.

## Methods

### Design

The current research was a cross-sectional from December 2021 to September 2022.

### Research location and sampling method

The population included all the nurses working in the inpatient wards of COVID-19 in the teaching hospitals of the university. The location was Shiraz, an educational hospital located in the south of Iran. Shiraz is the third largest city in Iran and has large educational hospitals with numerous inpatient departments. During the covid-19 era, elderly patients with covid-19 were hospitalized in four teaching hospitals of the university, including Namazi, Shahid Faghihi, Rajaei, and Hazrat Ali Asghar hospitals. Sampling method was Through census. Briefly, the names of all nurses in the COVID ward were taken from the nursing office of the province, and then they were included in the study based on the inclusion criteria (total 212 people).

### Inclusion and exclusion criteria

The inclusion criteria were nurses who had at least a bachelor’s degree in nursing. In addition, these nurses should be working in COVID-19 departments and have experience caring for elderly patients over 65 years old. Other nurses who completed the questionnaire incompletely or were transferred to other departments and hospitals were excluded from the study.

### Instruments

The data were collected through the questionnaire of demographic information and measuring the compassionate care of Tehranineshat and colleagues (2021).

### Demographic information

The questionnaire was based on age, sex, marital status, education level, work experience in the COVID-19 ward and work experience in the hospital, history of having a patient over 65 years old, history of having the necessary training to care for an elderly patient, and position of the nurse.

### Compassionate care assessment questionnaire

This questionnaire was compiled and psychometrically evaluated by Tehranineshat et al. (2021) based on 28 questions. The questionnaire items related to measuring nurses’ compassionate care are divided into four areas, including professional performance (9 items), continuous follow-up (6 items), patient-centered care (7 items), and empathic communication (6 items) based on a Likert scale (Always (5), often (4), sometimes (3), rarely (2) and never (1)). The overall score is obtained from the sum of the scores of the items. The duration of answering this questionnaire was about 5–10 min. The points obtained are defined based on the distance between the lower third of grades 28–65 (poor), the middle third of 66–103 (average) and the upper third of 104–140 (good). Psychometry of the questionnaire was performed by the researcher with validity and reliability. The validity of the questionnaire has been checked by examining face validity (qualitative-quantitative), content validity (qualitative-quantitative), construct validity using exploratory factor analysis, confirmatory factor analysis. In addition, the convergent validity of the correlation coefficient between the Compassionate Care Questionnaire and the Care Behavior Questionnaire of Wolf et al. was obtained at 0.68. The reliability of the questionnaire has been checked with internal consistency and stability methods. The reliability of the whole questionnaire was also estimated through Cronbach’s alpha coefficient (0.89) and intracluster correlation coefficient (0.94) [17].

### Ethics

The researcher went to the hospitals where the research was conducted after obtaining the license and code of ethics (IR.SUMS.NUMIMG.REC.1401.015) from the vice-chancellor for research and technology of the university. The purpose of the research was explained by the researchers to the officials of the respective hospitals and the samples were selected based on the inclusion criteria. Written informed consent was obtained from all samples, and the personal information of the participants was kept confidential. They were also assured that they information would remain confidential and they preserve the right for withdrawing from the study at any time they wished.

### Data analysis

The collected data were analyzed using descriptive and analytical methods. Qualitative data of the research study were described by frequency (percentage) and quantitative data by mean (standard deviation). Mann-Whitney test was used to investigate the relationship between nurses’ compassionate care and demographic characteristics in terms of a two-level variable, considering that normality was not established. Kruskal-Wallis’s test should be used for comparison in terms of multi-level variables due to non-establishment of normality. Based on the opinion of the statistical consultant, the imbalance in the number of multilevel variable samples made it sufficient to compare the average and describe visually. Spearman’s correlation test was used to measure the correlation of four variables of the target items (professional performance, continuous follow-up, patient-centered care and empathic communication) and quantitative variables regarding the non-establishment of normality The study utilized the generalized linear regression test to predict nurses’ scores based on demographic variables and dimensions of compassionate care. Analyzes were performed using SPSS software version 22.

## Results

Most of the participants in this study were women (86.3%), single people (50%), age group 20–30 years (54.7%), with a bachelor’s degree (95.3%). It was also found that most of the participants had the job position of nurse (92.9%). All the nurses in the study had at least one experience of nursing an elderly patient, but a high percentage of them (63.7%) did not receive special training regarding compassionate care of an elderly patient. According to the data analysis, 65.2% of the participants (135 people) have less than 10 years of work experience, and 39.9% of the nurses participating in the study (81 people) have been working in the COVID-19 ward for 1–2 years (Table 1).

**Table 1:** Demographic characteristics of research samples

Variable		Frequency	Frequency percentage
Gender	Female	183	86.3
	Male	29	13.7
Age	20–30 years	116	55.5
	30–40 years	65	31.1
	40–50 years	27	12.9
	Over 50 years old	1	0.5
Marital status	Single	106	50
	Married	102	48.1
	Divorced or widowed	4	1.9
Education	Bachelor	202	95.3
	Masters	10	4.7
	P.H.D	0	0
Nursing a patient over 65 years old	Positive	212	100
	Negative	0	0
Work experience in the COVID-19 department	Less than 1 year	72	35.5
	1–2 years	81	39.9
	More than 2 years	50	24.6
Hospital work experience	Less than 10 years	135	65.2
	10–20 years	66	31.9
	More than 20 years	6	2.9
Elderly care training course	Positive	77	36.3
	Negative	135	63.7
Position	Nurse	197	92.9
	Head nurse	15	7.1

The average score of nurses’ compassionate care in caring for the elderly in the covid-19 wards was 130.18 with a standard deviation of 9.42. In addition, the highest and lowest scores among the participants were 140 and 85, respectively, and the standard deviation was 9.42.

The relationship between each of the demographic variables and the nurses’ compassionate care score was investigated through Mann-Whitney and Spearman (Tables 2 and 3).

**Table 2:** Comparison of the mean score of compassionate care with qualitative demographic variables

Variable	Groups	Quantity	Mean	Standard deviation	Median	Significant level
Gender	Female	183	130.09	9.06	133(125–137)	0.31
	Male	29	130.72	11.61	135(125–138)	
Marital status	Single	110	129.93	9.47	133(123–138)	0.84
	Married	102	130.45	9.41	133(126.75–137)	
Education	Bachelor	202	130.26	9.389	133(126-137.25)	0.66
	Masters	10	128.50	10.544	130(120.5-137.5)	
Training course	Positive	77	130.87	8.855	133(125.5–138)	0.38
	Negative	135	129.79	9.748	132(125–137)	
Job position	Nurse	197	130	9.556	133(125–138)	0.95
	Head nurse	15	131.13	7.736	134(126–137)	

**Table 3:** Correlation between quantitative variables and compassion score

Variables	score	Work experience in the COVID-19 department	Hospital work experience	Age
**Score**	1			
Work experience in the COVID-19 department	r = 0.026 P = 0.717	1		
Hospital work experience	r = 0.092 P = 0.189		1	
**Age**	r = 0.204 P = 0.003			1

The score of compassionate care of nurses in caring for the elderly in the COVID-19 wards was analyzed by separating professional performance, continuous follow-up, patient-centered care, and empathic communication. The lowest average score was related to the empathic communication item and the highest average score was related to the professional performance item (Table 4).

**Table 4:** Compassionate care score of nurses based on the questionnaire dimensions

Dimension	Score			Standard deviation
	Min	Max	Mean	
Professional performance	27	45	43.179	2.799
Continuous follow-up	18	30	28.141	2.374
Patient-centered care	21	35	31.089	2.946
Empathic communication	16	30	27.768	2.970

To understand the relationship between the changes in the questionnaire and the demographic factors, the regression analysis was conducted. For this, the significance of each change was evaluated and only the variables with a significance level less than 0.2 were included in the final model used in the generalized linear model.

Based on the data, the final model included only age and work as predictors of professional performance. The results indicate that with each year increase in age, the performance score increases by 0.14. The R^2^ value of 0.06 suggests that age changes can explain about 6% of the variation in professional performance.

About continuous follow-up dimension,age and work experience were included in the final model, but neither of them were found to be statistically significant. Various variables were studied in relation to patient-centeredness and empathy in healthcare. Among these variables, age and education in caring for the elderly were included in the sample. The study found that only age had an effect on patient-centeredness, with an average increase of 0.07 points. With regard to empathy, only age was found to have a correlation with the score, but it was not statistically significant.

In addition, the study examined the relationship between the total score of compassionate care and demographic variables. The variables of age and work experience were included in the model, and the findings indicated that for every one year of age, the total score of compassionate care of nurses increased by 0.47 points. Various variables were studied in relation to patient-centeredness and empathy in healthcare. Among these variables, age and education in caring for the elderly were included in the sample. The study found that only age had an effect on patient-centeredness, with an average increase of 0.07 points. With regard to empathy, only age was found to have a correlation with the score, but it was not statistically significant.

Additionally, the study examined the relationship between the total score of compassionate care and demographic variables. The variables of age and work experience were included in the model, and the findings indicated that for every one year of age, the total score of compassionate care of nurses increased by 0.47 points (Table 5).

**Table 5:** The role of demographic variables in predicting nurses’ compassionate care score

Dimension	Demographic variables	β	SE	t	sig
Professional performance	Age	0.142	0.054	2.636	0.009
	Work year	−0.029	0.058	−0.498	0.619
Continuous follow-up	Age	0.074	0.047	1.579	0.116
	Work year	−0.017	0.050	−338	0.736
Patient-centered care	Training course	0.642	0.419	1.531	0.127
	Age	0.07	0.031	2.260	0.025
Empathic communication	Age	0.04	0.031	1.491	0.137
Score	Age	0.47	0.183	2.575	0.011
	Work year	−212	0.197	−1.074	0.284

## Discussion

According to the results, the average score of compassionate care is in the “good” range. The results of the present study were consistent with similar studies.In Kılıç et al.‘s study, the compassionate care score of nurses working in the COVID-19 ward shows a high level [18]. The average score of compassionate care was conducted in other studies before the pandemic and in wards other than COVID-19. For example, the score in Arkan et al., Arlı and Bakan, Büyük and Baltacı was high, and it can be said that the score of compassionate care of nurses after the epidemic was similar to their score before the pandemic [19–21]. The results of Nielsen et al. showed that the elderly patients who were hospitalized during the COVID-19 pandemic complained from painful experiences of lack of status or esteem, independence, and sense of self during the care. Meanwhile, the rules and restrictions resulting from COVID-19 had exacerbated the sense of loneliness and being left by the family members. Nurses also failed to care for all-round needs of elderly patients professionally [14]. The results of Hassan et al. showed that although nurses taking care of elderly patients had a positive attitude to compassionate care, their compassionate performance and self-confidence were low [22]. In the study by Simpson et al., it was found that compassionate care is not provided for those who have been hospitalized with COVID-19 diagnosis. According to the study participants’ views, sense of psychological security is essential for providing compassionate care, since compassion fatigue and burnout would affect the provision of compassionate care and quality of nursing care [23]. The patients in the study by Noor et al. stated that the period of COVID-19 pandemic had not affected compassionate care of nurses; even with restrictions on visits and strict regulations for care providers, most patients claimed that they had not been affected by changes in healthcare policies of the COVID-10 pandemic period [24]. The results of another study showed that during COVID-19 pandemic, healthcare staff provided care for patients with complete personal protective equipment including gown, gloves, face shield, and mask, and only the eyes of healthcare staff could be seen from below the mask or their glasses. Such restrictions resulting from isolation had created challenges for healthcare staff in their interaction with the patients and providing compassionate and quality care for patients. Nevertheless, compassion had remained the main core of care for some personnel. Patients with COVID-19 stated that nurses could express their compassion through simple measures such as establishing eye contact, response to the patient’s concerns, touching within the cultural norms, or tapping on the patient’s shoulder [25]. Although compassionate care should be provided in healthcare settings constantly, it can be affected by a complex range of personal and organizational factors. Provision of compassionate care results from inherent tendency to helping others within the framework of work-related goals and expectations. However, when it is applied to healthcare stings, its social aspect comes into play and forms as a salient feature of a professional community [26].

It was found that a considerable percentage of healthcare workers (63.7%) did not receive any special training on how to provide compassionate care to elderly patients. The data analysis revealed that 65.2% of the participants (135 individuals) had less than 10 years of work experience, while 39.9% of the nurses in the study (81 people) had been working in the COVID-19 ward for 1–2 years.

According to the findings of this study, the majority of nurses have not received any special training in providing compassionate care for elderly patients. The results of Babaei et al.‘s (2019) study also revealed that the in-service training programs failed to equip nurses with the necessary skills and knowledge for providing compassionate care, and they did not gain any such education during their academic studies. Nurses expressed their preference for training programs that would enhance their competency in the field of compassionate clinical care [27]. There is debate over whether compassion is an innate trait or can be learned [28, 29]. A healthcare professional’s capacity for empathetic care partially depends on their innate characteristics before entering the field [30]. But Professional motives, life experiences, individual, and organizational factors can strengthen or reduce these inherent characteristics [31, 32]. The inherent feature can also be influenced by cultural background and spiritual customs [33]. However, research suggests that this innate trait can be developed and often sustained through educational interventions such as experiential learning and reflective practices, as well as organizational support [28]. In some studies, nurses and other health professionals have proposed using clinical role-playing, rethinking, and simulation as educational tools to develop compassion [30, 34, 35]. In Jang et al.’s (2022) study, the “nursing services based on patient experience” intervention improved nurses’ compassion [36]. But many of the interventions that were implemented in the past did not address all aspects of compassionate care and were not founded on a model or definition of compassion. Therefore, it is recommended that educational programs for compassionate care should cover all necessary aspects including the development of attitudes, knowledge, skills, and behaviors based on compassion. Additionally, the effectiveness of nurse training should be assessed by patients, preceptors, and peers [31].

With regards to the dimensions of compassionate care provision in the present study, the highest mean score was related to the professional performance, and the lowest to the empathetic relationship. According to nurses in the study by Yodsuban et al., nurses should enjoy good professional as well as managerial knowledge and skills for taking care of elderly patients during COVID-19 pandemic. For reducing social isolation of elderly patients, they should employ holistic approach as well as all-round interventions with a focus on gratifying physical, psychological, and social needs of the elderly. [37]. In a study, patients with COVID 19 complained from insufficient interactions and lack of empathy during their communication. In order to establish empathetic as well as trust-based relationship with COVID-19 patients, the workplace institute had organized educational sessions about methods of verbal and nonverbal communication. In addition, a website was designed for follow-up of treatment and compassionate care for patients [38]. Sharing feelings and concerns causes all individuals involved in the healthcare crisis to find a greater sense of responsibility and adapt to the stressful conditions [39]. Empathy and effective communication are among the essential aspects of compassion-based care [28] and protective factors against emotional burnout of healthcare personnel [40]. Nevertheless, empathetic relationship is affected under stressful conditions such as COVID-19 and due to the personnel’s psychological and emotional burden and in turn their occupational burnout [41]. In addition, effective nurse-patient interaction is one of the important indicators of compassionate care that can be influenced by the culture of the community [42]. In Iranian communities, religion plays an important role in the quality of nursing care. Also, compliance with the religious and cultural norms of the community in the care of patients is also mandatory. For example, some loving behaviors such as touching the opposite sex are inappropriate and forbidden in Islam, and Muslim nurses should avoid such behaviors [43]. Compassionate care can be a basis for a comprehensive model in taking care of elderly with COVID-19, based on which in addition to typical context-based care of patients, nurses should also address concepts such as empathy, altruism, cultural values, and familial needs at the terminal stages of the patient’s life [44].

According to the study, compassion score and age were significantly correlated, although the relationship was weak. Arkan et al.’s study showed that age variable had a significant relationship with nurses’ compassionate care score [20]. The results of this study were in line with the current study. However, in the current study, the amount of this relationship was weak because the personnel in the COVID-19 ward was novice and they were passing their training course; thus, they homogeneous and young in terms of age and work experience. This issue could cause the lack of relationship between the variable of work experience and compassionate care score and the weak relationship between the variable of age and compassionate care score.

Other results were related to the examination of the compassionate care score by the dimensions of this type of care such as professional performance in which people scored high. A study on midwives showed a positive relationship between professional performance and compassion [45]. In a study, Bilgiç discussed the mutual relationship between these two variables and the results of the study indicated that increasing the level of compassion has a positive effect on moral sensitivity [46]. The result of this study was consistent with that of the present research. This topic shows the importance of professional ethics training in universities and hospitals and its effect on the behavior of nurses.

Another dimension of the questionnaire was the empathic relationship between the nurse and patient and investigated its relationship with the nurses’ compassionate care score. Jack stated that empathic communication is an integral aspect of caring for the elderly [47]. Fashami et al. concluded that compassionate care is the main component of empathy [48]. In addition, Su et al. proposed that compassionate care is a combination of the nurses’ empathic communication and their desire to reduce the suffering of patients, examine individual care needs, use effective communication with patients in line with their treatment and promote mutual benefits [49]. Therefore, the results of the study are related to the importance of empathic communication.

On the other hand, Generational differences between elderly people and nurses make establishing effective communication difficult and lead to poor quality care [50]. In the ethnographic study that was conducted in the southeast of Iran, it was found that communication problems between caregivers and the elderly are high. Thus, it is necessary that people who care for elderly individuals are trained in special communication strategies and skills, and they are not influenced by fatigue and burnout resulting from caring [51]. Strengthening communication skills and correcting the attitude of nurses towards elderly patients is one of the most important measures that can lead to the improvement of communication between the caregiver and the elderly [52].

Another dimension is the care follow-up questionnaire or continuity in care and its positive effect on the nurses’ compassionate care score. Jalalmarvi’s study showed that education and continuous care are among the main and important factors in the matter of care [53]. Hill and Freeman pointed out that continuity of care is one of the essential elements of modern care [54]. All the mentioned studies and other studies conducted in this field have focused on the importance of continuity in care or the follow-up of care in patient care, which was in line with the present study.

Patient-centered care is another dimension, which was investigated in the nurses’ compassionate care questionnaire. Ilarde et al. evaluated compassionate care in nurses and found that patients tend to feel valued and respected by health care providers. The patient-centered approach led to the facilitation of decision-making by patients and their families. Patient-centered care helped achieve care goals by determining the values and preferences of patients and their relatives. Involving patients and their families in care was one of the most important elements of compassionate care [55]. In another study, it is stated that compassionate and respectful care requires humanity and kindness to improve the quality of person-centered care for the patient [56]. Rawlings et al. stated that the patient should be considered as a partner in care planning and decision-making. The need for respecting the patients’ and their families’ desire to support them and allocate time for them was recognized as a necessary factor. A patient-centered approach to care facilitates the caregiver’s supportive role and facilitates the patient’s recovery process [57]. All studies studied have discussed the importance and emphasis of patient-centeredness in compassionate care, and thus, nurses should provide compassionate care with an emphasis on patient-centered care as a priority for health care.

was the lack of significant relationship between other demographic characteristics such as gender, education level, job history and category, marital status and nurses’ compassionate care score. Yilmaz-Esencan indicated that as midwives’ level of education decreased, their level of compassion also decreased and they received a lower score. Being married increased kindness and shared humanity as a factor influencing compassion [45]. In another similar study, Henderson emphasized the importance of the relationship between high education level and compassionate care [58]. In addition to these studies, a systematic review has been conducted and strong results indicate a positive effect of nurses’ education and level of education on the level of compassion in their care [59]. The results of the mentioned studies were not the same with the present study regarding the variable of education level. Perhaps this difference can be attributed to the low statistical population in the level of education above bachelor’s degree in the participants. Regarding the relationship between marital status and compassion score, the results of the Yilmaz-Esencan study were inconsistent with the present study, which could be due to the small number of participants [45].

Work history and job position were other demographic information that were examined in the present study to investigate their relationship with nurses’ compassionate care score. Lee et al. have also mentioned these variables and concluded that the compassion score of people who had 20 years of clinical experience was significantly higher compared to other people who had less clinical experience. In relation to the job position, this score was much higher in the manager of the nursing unit than nurses and clinical nursing specialists [60]. The results obtained from the present study regarding the effect of these two variables on the compassion score showed inconsistent results with Lee’s study and the reason for their difference. The current study was conducted on nurses working in the relevant departments during the epidemic, and most of the nurses working in the COVID-19 department included planned, novice, or contract personnel. The conditions were the same in relation to the job position variable.

Another variable that was examined in the current study was gender. A similar study was conducted in the United States and Ethiopia, in which the effect of gender and its relationship with the compassionate care score were investigated. The results of the research showed that the score of compassionate care is higher in women than in men [56, 61]. One of the results of a study conducted by Son on nursing interns was that female nurses scored higher in compassionate care than male nurses. This result was similar to Çingöl’s result on 494 nursing students to investigate the compassionate care score. Another researcher named Polat also conducted a similar study and the result of the study showed that the score of compassionate care is higher in women [62–64]. In the present study, the gender variable had no significant relationship with compassionate care of the patient and was inconsistent with the similar studies mentioned. Among the reasons, the low statistical population and the lack of distribution of the number of people in two groups can be mentioned. On the other hand, no information about the gender of the patients was available. In studies where women’s compassionate care scores were higher, usually most nurses are women, and since women are more emotionally sensitive and caring than men, women have higher levels of compassionate care. In some studies, such as Hanan Hassan’s study, which was mentioned earlier, no relationship was found between nurses’ gender and the compassionate care score [22]. This study is similar to the current study because no significant relationship was found between the gender variable and nurses’ compassion score.

### Limitations

It should be noted that over half of the nurses who participated in the study were young and inexperienced. This was due to the COVID-19 pandemic and the severe shortage of personnel, which forced the provincial nursing department to recruit young and inexperienced nurses to work in COVID-19 sections that were outside the researcher’s control. However, the researcher made sure to explain the objectives of the study to all participants before they completed the questionnaires.

Accordingly, it is suggested that in future studies, various age groups of nurses be examined. Also, the following issues can be considered for further research: exploring the compassionate care of nurses in other hospital wards, inspecting and comparing the compassionate care in nurses with other medical science professions, investigating the factors that impede compassionate care among nurses, exploring factors that cause improvements in the quality of compassionate care of nurses for patients, training self-compassion in nurses and examining its impact on nurses’ compassionate care, investigating compassionate care perceived by elderly hospitalized in COVID-19 wards with other wards of the hospital, scrutinizing different age group communities of nurses with regards to their compassionate care score.

## Conclusions

Based on the results, the average score of compassionate care of nurses participating in the study is generally at a good level during COVID-19. Professional performance, continuity of care, patient-centeredness, and empathic communication were manifestations of compassionate care. Therefore, nurses should be trained to improve their performance and enhance the patients’ treatment process, especially in the elderly group, who make up a large part of the patients admitted to the hospital. Also, in-service training programs should be widely applied for nurses so that they can expand their knowledge and performance with regards to compassionate care especially during pandemics.

## Data Availability

The datasets during the current study are not publicly available due to confidentiality of the nurses’ data, but it will be made available upon reasonable request from the corresponding author.

## Abbreviations

SPSS: Software package used for Statistical Analysis
